# Risk factors for impulse control disorders and related behaviors in Parkinson’s disease: secondary analyses of the ICARUS study

**DOI:** 10.1080/21556660.2019.1675670

**Published:** 2019-10-04

**Authors:** Paolo Barone, Angelo Antonini, Paolo Stanzione, Karin Annoni, Mahnaz Asgharnejad, Ubaldo Bonuccelli

**Affiliations:** a Department of Medicine, Center for Neurodegenerative Diseases (CEMAND), University of Salerno, Baronissi, Italy;; b Department of Neurosciences (DNS), Padova University, Padova, Italy;; c Department of Systems Medicine, University of Rome Tor Vergata, Rome, Italy;; d IRCCS Fondazione S Lucia, Rome, Italy;; e UCB Pharma, Milan, Italy;; f UCB Pharma, Raleigh, NC, USA;; g Department of Clinical and Experimental Medicine, Neurology Unit, University of Pisa, Pisa, Italy

**Keywords:** Impulse control disorders, incidence, Parkinson’s disease, risk factors, predictive factors, ICARUS study

## Abstract

**Objective:** Impulse control disorders and related behaviors (ICDs) are common in patients with Parkinson’s disease (PD), yet incidence and predictive factors are not fully understood. We examined the epidemiology of ICDs in PD through secondary and *post-hoc* analyses of data from the ICARUS (SP0990) study, which enrolled >1000 patients.

**Methods:** Using a modified-Minnesota Impulsive Disorders Interview (mMIDI), ICD incidence was calculated for patients who were ICD-negative at baseline but ICD-positive at year 1, and year 1 and/or 2 (cumulative 2-year ICD incidence). The proportion of “new cases” (ICD-negative at baseline, but ICD-positive at year 1 or 2), and “remitters” (ICD-positive at baseline but ICD-negative at year 1 or 2) was also calculated for the whole ICARUS population.

**Results:** Among 709 patients ICD-negative at baseline, 97 screened ICD-positive (13.7%) at year 1. Among 712 patients who were ICD-negative at baseline, 147 were ICD-positive at ≥1 post-baseline visit (20.6%). Among patients who were ICD-negative at baseline who subsequently experienced an ICD, a higher proportion were male or smokers, younger at baseline, younger at disease/symptom onset, and had longer disease duration. Among the whole population, a similar proportion were “new cases” at years 1 (9.7%) and 2 (8.6%) versus the previous visit. The proportion of “remitters” was slightly higher at year 2 (11.0%) than 1 (9.1%) versus previous visit.

**Conclusions:** The proportion of ICD-remitters approximately matched/exceeded new cases, suggesting patients with ICD are in a state of flux. Current data allow for a conservative estimate of 2-year ICD incidence in ICARUS of ∼21% of patients, not accounting for transient new ICD cases between visits.

## Introduction

Over the past two decades, impulse control disorders and other related behaviors (altogether hereafter referred to as “ICD behaviors” or “ICDs”) have been recognized as a relatively common comorbidity in patients with Parkinson’s disease (PD)^1–5^. In the published ICARUS (Impulse Control disorders And the association of neuRopsychiatric symptoms, cognition and qUality of life in ParkinSon disease; SP0990) study, one of the largest prospective studies of ICDs in PD to date, the *point prevalence* of ICDs was relatively stable at 26.5 to 29.3% across the 2-year observation period^6^.

ICDs are most likely to develop as a result of dopaminergic medication use^7^, with a number of other risk factors identified, such as younger age, motor complications, depression, family history of ICDs, alcohol use, nicotine dependence, and certain personality traits^3^^,^^8–11^. Research also suggests that people with PD with specific frontal dysfunctions may be more likely to develop an ICD when taking anti-Parkinson’s medication^12^. In the ICARUS study, patients who were ICD-positive at study baseline had more severe non-motor symptoms (including mood and sexual function) and depression, as well as poorer sleep quality and reduced PD-related quality of life compared with those who were ICD-negative^6^.

The *incidence* of ICDs among people with PD is not consistently reported. One single-site study, conducted in small sample size, reported a cumulative ICD incidence of 39.1% during 21 months of dopamine agonist (DA) treatment in PD patients with no previous ICDs; cigarette smoking, caffeine use, motor complications, and higher peak DA use were identified as risk factors^11^. A more recent analysis of data from 320 early-stage PD patients with no prior ICDs from the Parkinson Progression Markers Initiative (PPMI) database reported cumulative incidence of 8% (year 1), 18% (year 2), and 25% (year 3) post-baseline^13^. Younger age at baseline was a risk factor for incident ICD symptoms, while sex, education, and baseline global cognitive performance, anxiety symptoms, depressive symptoms, and motor severity were not significantly associated with incident ICD symptoms^13^.

The natural history of ICDs in PD is not clearly established, and few studies report the long-term outcome of interventions for ICDs in PD. Among 12 patients with PD who had discontinued or significantly decreased DA treatment in one long-term follow-up study, 10 (83%) no longer met ICD diagnostic criteria after a mean follow-up period of 29 months^14^. However, ICDs may sometimes be resistant to dopaminergic medication reduction^15^.

In order to minimize this complication and help guide treatment decisions, it is important to further understand the incidence and predictive factors for ICDs along the disease journey. Here, we examine the epidemiology of ICDs in PD through secondary and *post-hoc* analyses of data from more than 1000 Italian outpatients enrolled in the prospective ICARUS study, by reporting:

- Incidence at year 1 and cumulative 2-year ICD incidence;
- Risk factors for ICD development (overall and individual ICD subtypes) among patients who were ICD-negative at study baseline;
- Proportion of new ICD cases and ICD remissions (overall and individual ICD subtypes) at year 1 and year 2 among the whole ICARUS population. Note that an analysis of new ICD and ICD remissions by baseline ICD subtype was not of interest at the time these *post-hoc* analyses were performed.

## Methods

### Study design

ICARUS was a prospective, non-interventional, multicenter study in treated Italian outpatients with PD. A detailed description of the ICARUS study design has been published previously^6^. The primary variable was the presence (prevalence and incidence) of overall ICDs and ICD subtypes according to a modified version of the Minnesota Impulsive Disorders Interview (mMIDI)^16^. ICD status was assessed at three study visits: baseline, year 1, and year 2. Switching of patient treatment was permitted at any time during the study, at the discretion of the treating physician.

### Measurements

A patient was considered ICD-positive if they answered affirmatively at the mMIDI scale to one gateway question and to one or more of the remaining questions in the same ICD module of the mMIDI interview.

### Estimates of ICD incidence

In addition to the point prevalence of ICDs (reported in the primary publication^6^), the planned primary analysis included ICD incidence calculations. However, as ICD presence was provided for specific time points (i.e. the three visits) and not time periods, the true incidence could not be calculated. For the current analysis, we have therefore calculated “conservative” incidence, with the caveat that transient new ICD cases occurring within the year between visits have remained unrecorded, thus underestimating the true incidence.

Using the Full Analysis Set^6^, ICD incidence reported in this analysis was calculated for patients who were ICD-negative at baseline, but were positive for an ICD by mMIDI (Table 1) at:

**Table 1.** Definition of year 1 incidence and cumulative 2-year incidence.

Year 1 incidence		
Baseline	Year 1	
–	+	
Cumulative 2-year incidence		
Baseline	Year 1	Year 2
–	–	+
–	+	–
–	+	+

Abbreviations. ICD, impulse control disorder; –, ICD-negative; +, ICD-positive.

- Year 1 (conservative estimate of ICD incidence for year 1);
- Year 1 and/or 2 (conservative estimate of cumulative 2-year ICD incidence).

The number of patients who were ICD-negative at baseline was used as the denominator.

### *Post-hoc* analyses of baseline data according to subsequent ICD status

The large number of patients involved in the ICARUS study permitted meaningful *post-hoc* examination of baseline data for the subgroup of patients who were negative for ICD at baseline. Patients in this subgroup were further subdivided into “ICD-positive after baseline” (patients who were positive for an ICD at the year 1 and/or year 2 study visits) and “ICD-negative after baseline” (patients who were negative for an ICD at year 1 and year 2 study visits) to identify baseline characteristics that were different between the groups, including:

- Gender, age at baseline, age at PD onset/symptom onset, PD duration, smoking status, alcohol consumption, education level, marital status, employment status, early discontinuation reason, and region of Italy;
- Disease status according to:
  1. PD treatment,
  2. Functional disability (Hoehn & Yahr [HY]),
  3. Cognitive function (Mini-Mental State Examination [MMSE], Frontal Assessment Battery [FAB], Parkinson’s Disease-Cognitive Rating Scale [PD-CRS]),
  4. Beck Depression Inventory-II (BDI-II),
  5. Parkinson’s Disease Non-Motor Symptom Scale (PD-NMSS),
  6. Parkinson’s Disease Questionnaire-8 item short form (PDQ-8),
  7. Parkinson’s Disease Sleep Scale-2 (PDSS-2).

### Shift in ICD status: “new cases” and “remitters”

In addition to conservative incidence, we report the number and proportion of “new cases” at year 1 relative to baseline, at year 2 relative to year 1, and at year 2 relative to baseline (the latter irrespective of ICD status at year 1). In recognition that ICD status may also reverse, we also report the number and proportion of “remitters” at year 1 relative to baseline, at year 2 relative to year 1, and at year 2 (the latter irrespective of ICD status at year 1) (Table 2).

**Table 2.** Definition of shift in ICD status at year 1 versus baseline and year 2 versus year 1 or baseline.

ICD status at year 1				
Baseline	Year 1	Comparison: Year 1 vs. baseline		
–	+	New case		
+	–	Remitter		
–	–	Negative unchanged		
+	+	Positive unchanged		
ICD status at year 2				
Baseline	Year 1	Year 2	Comparison: year 2 vs. year 1	Comparison: year 2 vs. baseline
–	–	+	New case	New case
–	+	–	Remitter	Negative unchanged
–	+	+	Positive unchanged	New case
–	–	–	Negative unchanged	Negative unchanged
+	+	–	Remitter	Remitter
+	–	+	New case	Positive unchanged
+	–	–	Negative unchanged	Remitter
+	+	+	Positive unchanged	Positive unchanged

Abbreviations. ICD, impulse control disorder; –, ICD-negative; +, ICD-positive.

The proportion of “new cases” and “remitters” was calculated as the percentage of the total number of patients assessed at a given visit (i.e. both ICD-positive and -negative).

New cases and remitters were also summarized by baseline characteristics, including gender, age at baseline, age at PD onset, PD duration, and disease status according to PD treatment.

All data were summarized descriptively.

## Results

### ICD incidence/”new cases”

Among the 709 patients who were ICD-negative at baseline and had year 1 ICD data, 97 screened ICD-positive at year 1, resulting in a conservative estimate for ICD incidence for year 1 of 13.7%.

There were 712 patients who were ICD-negative at baseline with at least one post-baseline visit. Among them, 147 screened positive for an ICD at least at one post-baseline visit (i.e. at year 1, year 2, or both), resulting in a conservative estimate for cumulative 2-year ICD incidence of 20.6%.

### Analysis of risk factors for “new cases”

Among patients ICD-negative at baseline, there was a higher proportion of males who subsequently experienced an ICD. Moreover, patients who subsequently experienced an ICD were younger at baseline, younger at disease and symptom onset, had a longer disease duration, and a greater proportion were smokers compared with those who did not develop an ICD after baseline (Table 3).

**Table 3.** Baseline characteristics of patients negative for ICD at baseline according to subsequent ICD status at either post-baseline visit (FAS).

	ICD after baseline	No ICD after baseline
Patients	*N* = 147 (22%)	*N* = 521 (78%)
Males	71.4%	58.0%
Age (years)	64.3 (9.1)	67.1 (9.2)
BMI (kg/m^2^)	26.4 (3.8)	25.7 (3.5)
Female		
Age (years)	65.6 (9.7)	66.8 (9.6)
BMI (kg/m^2^)	25.5 (4.1)	25.4 (4.0)
Male		
Age (years)	63.8 (8.9)	67.3 (9.0)
BMI (kg/m^2^)	26.8 (3.6)	26.0 (3.1)
Age at PD onset (years)	57.6 (10.5)	61.8 (10.2)
Age at PD symptom onset (years)	56.8 (11.0)^a^	60.8 (10.5)^b^
Duration of PD (years)	6.7 (5.7)	5.3 (4.4)
Side of PD onset		
Right	51.7%	56.6%
Left	48.3%	42.6%
Not specified	0%	0.8%
PD symptom severity		
Hoehn & Yahr	2.0 (0.6)	1.9 (0.6)
PD-NMSS	29.9 (25.3)	31.5 (31.1)
PDSS-2	13.9 (9.9)	11.3 (8.4)^c^
PDQ-8	22.4 (15.4)	19.9 (15.6)
BDI-II	10.0 (7.5)^d^	8.8 (7.4)^e^
MMSE	28.6 (1.5)	28.6 (1.6)
PD-CRS	82.0 (19.3)	81.3 (19.2)^f^
FAB	14.9 (3.1)	15.0 (2.8)
Region of Italy		
North West	24.5%	22.5%
North East	18.4%	14.0%
Center	22.4%	29.4%
South	23.1%	17.1%
Islands	11.6%	17.1%
Discontinuation, *n*		
Use of excluded drugs	1	0
Lost to follow-up	6	0
Other	3	1
Smoker	14.3%	8.1%
Alcohol consumption		
No alcohol	42.9%	46.3%
Occasional consumption	43.5%	40.5%
Regular consumption	13.6%	13.2%
Education		
No/incomplete	2.7%	2.9%
University degree	16.3%	13.2%
Marital status		
Married	82.3%	82.9%
Unmarried	3.4%	3.5%
Widow	6.8%	10.2%
Other	7.5%	3.5%
Employment		
Retired	65.3%	67.9%
Housewife	6.1%	10.6%
Unemployed	1.4%	1.3%
Other^g^	27.2%	20.2%

Abbreviations. ICD, impulsive control disorder; FAS, full analysis set; BMI, body mass index; PD, Parkinson’s disease; MMSE, Mini-Mental State Examination; PD-NMSS, Parkinson’s Disease Non-Motor Symptom Scale; PDSS-2, Parkinson’s Disease Sleep Scale-2; PD-CRS, Parkinson’s Disease-Cognition Rating Scale; PDQ-8, Parkinson’s Disease Questionnaire-8 item short form; BDI-II, Beck Depression Inventory-II; FAB, Frontal Assessment Battery.

Data are mean (SD) unless otherwise specified.

a *n* = 143; ^b^*n* = 515; ^c^*n* = 520; ^d^*n* = 145; ^e^*n* = 517; ^f^*n* = 516; ^g^Includes freelancer, teacher, clerk, workman, trader, craftsman, and other; for the “no ICD after baseline” group, this category also includes manager and farmer.

Baseline severity of PD symptoms and functional disability (HY stage), cognitive function (MMSE, FAB, and PD-CRS), and non-motor symptoms (PD-NMSS total score) were similar between patients who did and did not develop an ICD after baseline (Table 3). However, those who did develop an ICD after baseline had slightly worse depressive symptoms (BDI-II), PD-related health status (PDQ-8), and sleep (PDSS-2) impairment (Table 3).

### ICD status: “new cases” and “remitters”

**Figure 1.**
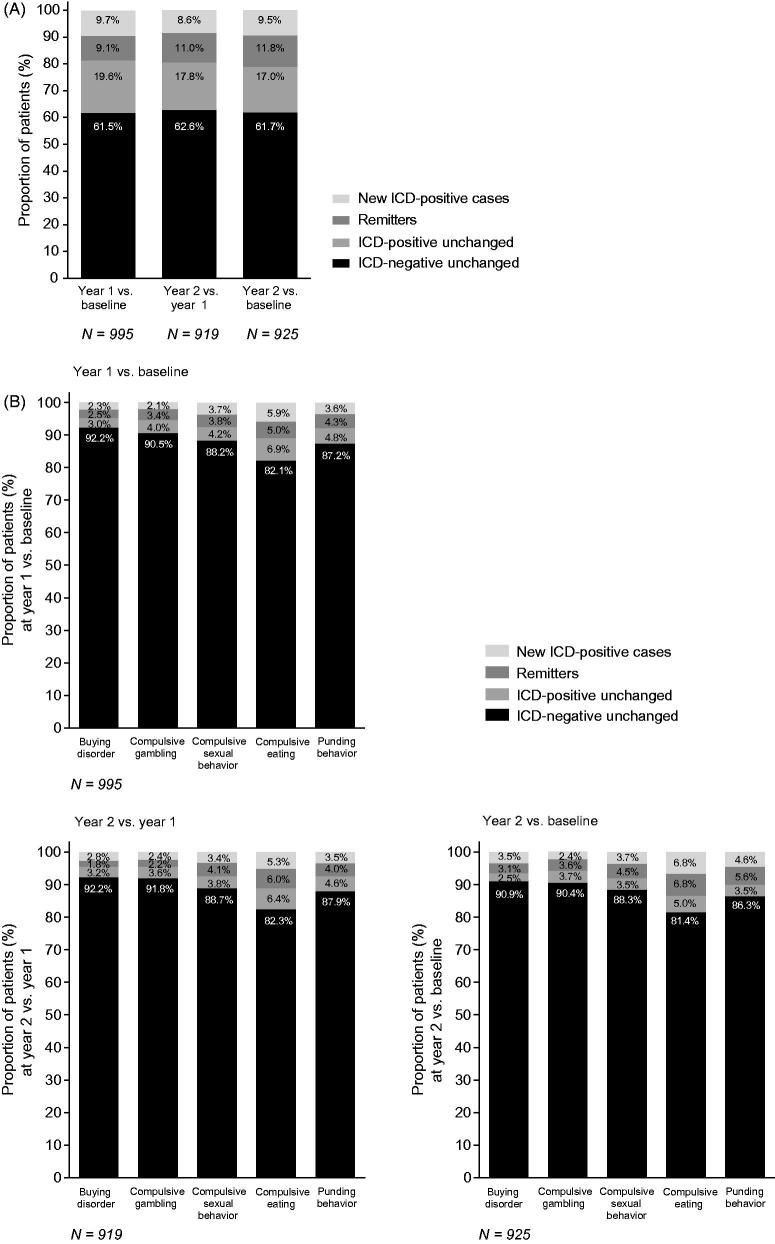
ICD status (“new cases” and “remitters”) by modified MIDI, (A) overall ICD behaviors and (B) ICD behavior subtypes (FAS). Abbreviations. ICD, impulsive control disorder; MIDI, Minnesota Impulsive Disorders Interview; PD, Parkinson’s disease.

Among the whole population, a similar proportion of patients were considered “new cases” at year 1 (9.7%) versus baseline and year 2 (8.6%) versus year 1 (Figure 1(A)). The proportion of “remitters” was slightly higher at year 2 versus year 1 (11.0%) compared with year 1 versus baseline (9.1%) (Figure 1(A)). A similar pattern was seen when comparing year 2 and baseline (Figure 1(A)). The proportion of “new cases” and “remitters” was highest for compulsive eating; however, there was no obvious difference between the ICD subtypes in the proportion of “new cases” versus “remitters” (Figure 1(B)).

### ICD status: demographic/clinical features

**Figure 2.**
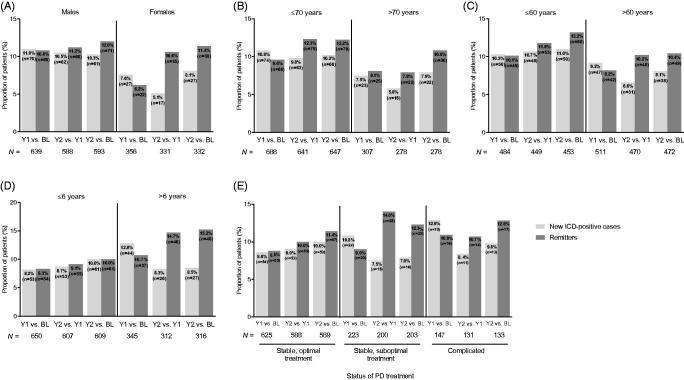
ICD status (“new cases” and “remitters”) by: (A) gender, (B) age at baseline, (C) age at PD onset, (D) PD duration at baseline, and (E) disease status according to PD treatment at baseline. Abbreviations. BL, baseline; ICD, impulsive control disorder; MIDI, Minnesota Impulsive Disorders Interview; PD, Parkinson’s disease; Y, year.

Analysis of ICD status by mMIDI for selected patient demographic and clinical features indicated some differences in the frequency of “new cases” (Figure 2). Being male, younger and having had the onset of PD at an earlier age indicated higher frequencies of “new cases” (Figures 2(A–C)).

The number of “remitters” frequently matched or exceeded the number of “new cases”.

### ICD status: ICD behavior subtypes by demographic/clinical features

Examination of ICD behavior subtypes according to demographic and clinical features at baseline among those with shifted ICD status (Table 4) indicated that generally, the numbers of “new cases” and “remitters” were similar or there were more remitters.

**Table 4.** ICD subtype status (“new cases” and “remitters”) at post-baseline visits by demographic and clinical features at baseline (FAS).

		Buying disorder		Compulsive gambling		Compulsive sexual behavior		Compulsive eating		Punding behavior	
	*N*	New cases	Remitters	New cases	Remitters	New cases	Remitters	New cases	Remitters	New cases	Remitters
Year 1 vs. baseline											
Gender											
Males	639	19 (3.0%)	18 (2.8%)	14 (2.2%)	23 (3.6%)	34 (5.3%)	34 (5.3%)	39 (6.1%)	29 (4.5%)	27 (4.2%)	31 (4.9%)
Females	356	4 (1.1%)	7 (2.0%)	7 (2.0%)	11 (3.1%)	3 (0.8%)	4 (1.1%)	20 (5.6%)	21 (5.9%)	9 (2.5%)	12 (3.4%)
Age at baseline											
≤70 years	688	19 (2.8%)	21 (3.1%)	19 (2.8%)	28 (4.1%)	33 (4.8%)	29 (4.2%)	44 (6.4%)	37 (5.4%)	26 (3.8%)	37 (5.4%)
>70 years	307	4 (1.3%)	4 (1.3%)	2 (0.7%)	6 (2.0%)	4 (1.3%)	9 (2.9%)	15 (4.9%)	13 (4.2%)	10 (3.3%)	6 (2.0%)
Age at PD onset											
≤60 years	484	14 (2.9%)	18 (3.7%)	11 (2.3%)	27 (5.6%)	21 (4.3%)	17 (3.5%)	34 (7.0%)	28 (5.8%)	20 (4.1%)	26 (5.4%)
>60 years	511	9 (1.8%)	7 (1.4%)	10 (2.0%)	7 (1.4%)	16 (3.1%)	21 (4.1%)	25 (4.9%)	22 (4.3%)	16 (3.1%)	17 (3.3%)
PD duration											
≤6 years	650	12 (1.8%)	13 (2.0%)	12 (1.8%)	13 (2.0%)	19 (2.9%)	24 (3.7%)	34 (5.2%)	27 (4.2%)	19 (2.9%)	29 (4.5%)
>6 years	345	11 (3.2%)	12 (3.5%)	9 (2.6%)	21 (6.1%)	18 (5.2%)	14 (4.1%)	25 (7.2%)	23 (6.7%)	17 (4.9%)	14 (4.1%)
Disease status (PD treatment)											
Stable, optimal treatment	625	13 (2.1%)	15 (2.4%)	11 (1.8%)	15 (2.4%)	24 (3.8%)	21 (3.4%)	26 (4.2%)	27 (4.3%)	21 (3.4%)	25 (4.0%)
Stable, suboptimal treatment	223	5 (2.2%)	5 (2.2%)	6 (2.7%)	11 (4.9%)	6 (2.7%)	13 (5.8%)	23 (10.3%)	10 (4.5%)	6 (2.7%)	8 (3.6%)
Complicated	147	5 (3.4%)	5 (3.4%)	4 (2.7%)	8 (5.4%)	7 (4.8%)	4 (2.7%)	10 (6.8%)	13 (8.8%)	9 (6.1%)	10 (6.8%)
Year 2 vs. year 1											
Gender											
Males	588	20 (3.4%)	11 (1.9%)	21 (3.6%)	13 (2.2%)	28 (4.8%)	35 (6.0%)	40 (6.8%)	31 (5.3%)	22 (3.7%)	27 (4.6%)
Females	331	6 (1.8%)	6 (1.8%)	1 (0.3%)	7 (2.1%)	3 (0.9%)	3 (0.9%)	9 (2.7%)	24 (7.3%)	10 (3.0%)	10 (3.0%)
Age at baseline											
≤70 years	641	24 (3.7%)	15 (2.3%)	20 (3.1%)	17 (2.7%)	23 (3.6%)	33 (5.1%)	40 (6.2%)	43 (6.7%)	25 (3.9%)	30 (4.7%)
>70 years	278	2 (0.7%)	2 (0.7%)	2 (0.7%)	3 (1.1%)	8 (2.9%)	5 (1.8%)	9 (3.2%)	12 (4.3%)	7 (2.5%)	7 (2.5%)
Age at PD onset											
≤60 years	449	19 (4.2%)	13 (2.9%)	16 (3.6%)	8 (1.8%)	18 (4.0%)	23 (5.1%)	35 (7.8%)	31 (6.9%)	18 (4.0%)	19 (4.2%)
>60 years	470	7 (1.5%)	4 (0.9%)	6 (1.3%)	12 (2.6%)	13 (2.8%)	15 (3.2%)	14 (3.0%)	24 (5.1%)	14 (3.0%)	18 (3.8%)
PD duration											
≤6 years	607	13 (2.1%)	9 (1.5%)	13 (2.1%)	12 (2.0%)	19 (3.1%)	20 (3.3%)	30 (4.9%)	28 (4.6%)	19 (3.1%)	19 (3.1%)
>6 years	312	13 (4.2%)	8 (2.6%)	9 (2.9%)	8 (2.6%)	12 (3.8%)	18 (5.8%)	19 (6.1%)	27 (8.7%)	13 (4.2%)	18 (5.8%)
Disease status (PD treatment)											
Stable, optimal treatment	588	13 (2.2%)	9 (1.5%)	15 (2.6%)	13 (2.2%)	21 (3.6%)	22 (3.7%)	25 (4.3%)	27 (4.6%)	18 (3.1%)	23 (3.9%)
Stable, suboptimal treatment	200	8 (4.0%)	5 (2.5%)	6 (3.0%)	4 (2.0%)	7 (3.5%)	10 (5.0%)	12 (6.0%)	22 (11.0%)	9 (4.5%)	9 (4.5%)
Complicated	131	5 (3.8%)	3 (2.3%)	1 (0.8%)	3 (2.3%)	3 (2.3%)	6 (4.6%)	12 (9.2%)	6 (4.6%)	5 (3.8%)	5 (3.8%)
Year 2 vs. baseline											
Gender											
Males	593	24 (4.0%)	18 (3.0%)	19 (3.2%)	21 (3.5%)	30 (5.1%)	38 (6.4%)	50 (8.4%)	35 (5.9%)	29 (4.9%)	36 (6.1%)
Females	332	8 (2.4%)	11 (3.3%)	3 (0.9%)	12 (3.6%)	4 (1.2%)	4 (1.2%)	13 (3.9%)	28 (8.4%)	14 (4.2%)	16 (4.8%)
Age at baseline											
≤70 years	647	29 (4.5%)	25 (3.9%)	20 (3.1%)	26 (4.0%)	28 (4.3%)	33 (5.1%)	49 (7.6%)	50 (7.7%)	31 (4.8%)	45 (7.0%)
>70 years	278	3 (1.1%)	4 (1.4%)	2 (0.7%)	7 (2.5%)	6 (2.2%)	9 (3.2%)	14 (5.0%)	13 (4.7%)	12 (4.3%)	7 (2.5%)
Age at PD onset											
≤60 years	453	22 (4.9%)	21 (4.6%)	16 (3.5%)	25 (5.5%)	21 (4.6%)	22 (4.9%)	43 (9.5%)	40 (8.8%)	23 (5.1%)	30 (6.6%)
>60 years	472	10 (2.1%)	8 (1.7%)	6 (1.3%)	8 (1.7%)	13 (2.8%)	20 (4.2%)	20 (4.2%)	23 (4.9%)	20 (4.2%)	22 (4.7%)
PD duration											
≤6 years	609	18 (3.0%)	16 (2.6%)	14 (2.3%)	13 (2.1%)	22 (3.6%)	24 (3.9%)	40 (6.6%)	32 (5.3%)	26 (4.3%)	33 (5.4%)
>6 years	316	14 (4.4%)	13 (4.1%)	8 (2.5%)	20 (6.3%)	12 (3.8%)	18 (5.7%)	23 (7.3%)	31 (9.8%)	17 (5.4%)	19 (6.0%)
Disease status (PD treatment)											
Stable, optimal treatment	589	17 (2.9%)	16 (2.7%)	13 (2.2%)	16 (2.7%)	26 (4.4%)	25 (4.2%)	33 (5.6%)	35 (5.9%)	24 (4.1%)	33 (5.6%)
Stable, suboptimal treatment	203	10 (4.9%)	8 (3.9%)	7 (3.4%)	9 (4.4%)	5 (2.5%)	13 (6.4%)	18 (8.9%)	16 (7.9%)	11 (5.4%)	11 (5.4%)
Complicated	133	5 (3.8%)	5 (3.8%)	2 (1.5%)	8 (6.0%)	3 (2.3%)	4 (3.0%)	12 (9.0%)	12 (9.0%)	8 (6.0%)	8 (6.0%)

Abbreviations. ICDL, impulsive control disorder; FAS, full analysis set; PD, Parkinson’s disease.

The gray fields = difference of ≥2% between “new cases” and “remitters”.

## Discussion

The substitute method for ICD incidence calculation in the ICARUS study suggests a 1-year incidence of approximately 14% on average, with a conservative 2-year cumulative incidence of approximately 21%. These conservative incidences are lower than those reported in a previous study (cumulative 21-month incidence of 39.1%)^11^, but similar to those reported in the PPMI cohort in patients with early-stage PD with no previous ICDs^13^. However, the values in our study underestimate the true incidence, given that transient new ICD cases occurring within the year between visits would have remained unrecorded.

The frequency of “new cases” and “remitters” at each visit was also calculated among the whole PD population of the ICARUS study. Whilst the 1-year risk of ICD development among the ICD-negative patients was ∼14%, a new ICD case occurred in ∼9% of patients each year when considering the whole population (ICD-positive and -negative, regardless of remission status). On average, 10% of patients remitted in a year (9.1% at year 1 vs. baseline and 11.0% at year 2 vs. year 1).

Given that the prevalence of ICDs in the ICARUS study was relatively stable at an average of 28% across the three study visits^6^, observed fluctuation of “new cases” and “remitters” suggests that ICDs in PD may be sensitive to treatment adjustments or other factors including dyskinesia^17^; this remains speculative as no such analysis was performed. However, ICD has been shown to peak 4.5 to 5 years after PD treatment initiation^18^. “Remitters” could represent change due to treatment adjustments (either removal of a drug with adverse event or improvement from treatment), as a result of higher disease awareness in the literature and thus better treatment management. Interestingly, among patients who were defined as suboptimal or with complicated PD at baseline, there were more new ICD cases than remitters at year 1; however, at year 2, the pattern was reversed, with more remitters than new ICD cases, which may reflect an initial non-efficient treatment schedule and a subsequent change in treatment strategy (as described above) in these patients. Indeed, the ALTHEA (Italian vALidation of THe unifiEd dyskinesiA rating scale) study showed that dopaminergic therapy total dose is associated with ICD severity and that patients with maladaptive behaviors and dyskinesia should be carefully evaluated because clinicians do not properly assess their motor and non-motor status^17^. The current study could not address these aspects because medication data were not collected at post-baseline time points.

Generally, the estimates of incidence from the current analysis support previous associations with demographic/clinical features observed in ICARUS^6^ and previous research^3^ for prevalence data in a broad patient population. In relation to risk factors for incident ICDs, in the current analysis, younger age was associated with a greater risk, as was also reported in the PPMI cohort^13^. In addition, consistent with previous research^11^, smoking at baseline was associated with a greater risk of new-onset ICDs. Being male, having depressive symptoms, and sleep impairment (as per PDSS-2, which measures sleep disturbances due to motor and non-motor symptoms at night, and overall quality of sleep), were found to increase the risk for new-onset ICDs in the current study, unlike in the previous studies examining risk factors for incident ICDs^11^^,^^13^. Cognitive performance was not predictive of ICD development in the current analysis, consistent with the prior studies, which either showed that global cognitive performance was not significantly associated with incident ICD symptoms^13^, or the absolute difference between ICD-positive and ICD-negative groups was small^11^. Some differences in findings across these studies maybe because of the different instruments used to measure PD symptom severity and differences in the methods used to assess risk factors.

This analysis has some limitations. The primary variable of the ICARUS study was the presence (prevalence and incidence) of overall ICDs and ICD subtypes according to mMIDI. Notably, this could be regarded as a surrogate primary variable because the Diagnostic and Statistical Manual of Mental Disorders, 4th Edition (DSM-IV), was not consistently used for ICD confirmation (as originally planned in the protocol); even so, DSM-IV does not cover all ICD subtypes that occur in patients with PD. Secondly, ICD status was assessed at three study visits (baseline, year 1, and year 2) and not throughout the study period, meaning ICD status between study visits was unknown. Thirdly, treatment could be switched at any time and this was not recorded, which may have influenced the number of “remitters”, with potentially a considerable lag phase of months to years. Finally, some of the findings are based on *post-hoc* analysis of small numbers of patients (in the 10 s) relative to the original 1000 patients recruited, increasing variation as a result of chance.

## Conclusion

Among PD patients in the ICARUS study, the number of ICD remitters approximately matched or exceeded new cases, suggesting patients with ICD are in flux and the state is variable. A conservative estimate of the cumulative incidence of ICD among PD patients in ICARUS was 21% of patients over the 2-year period. This underestimates the true incidence as it does not account for the transient new ICD cases occurring within the years between visits. It remains a possibility that the numbers of remitters are a result of treatment adjustments. This observational study containing a broad patient population highlights the importance of closely monitoring patients for ICD-type behaviors throughout the disease course and represents an interesting area for future research.

## Transparency

### Declaration of financial/other relationships

PB, AA, PS, and UB were study investigators on ICARUS, a UCB Pharma-sponsored study. PB has received personal fees from Acorda Therapeutics, UCB Pharma, and Zambon, and grants from AbbVie, Biotie Therapies, and Zambon. AA has received consultancy fees/honoraria from AbbVie, UCB Pharma, Zambon, Angelini, Lundbeck, Mundipharma, and Medtronic; has served on advisory boards for AbbVie and Acadia; provided expert testimony for Boehringer Ingelheim (pathological gambling cases); and received grants from Neureca Foundation, Gossweiler Foundation, Mundipharma, Italian National Research (project numbers RF-2009-1530177 and RF-2010-2319551), and Horizon2020 (project number 643706). PS has received consultancy fees/honoraria from UCB Pharma, Zambon, and Chiesi, and has served on advisory boards for UCB Pharma and Italian National Health Minister Research (project number RF-2013-2719661). UB has received personal compensation for serving on scientific advisory boards from UCB Pharma and Zambon. KA is a salaried employee of UCB Pharma. MA is a former employee of UCB Pharma and received stock options from her employment. JDA peer reviewers on this manuscript have no relevant financial or other relationships to disclose.
